# Supplementary material for: Reaching the Tumor: Mobility of Polymeric Micelles Inside an In Vitro Tumor-on-a-Chip Model with Dual ECM
Source: ACS Appl Mater Interfaces. 2023 Dec 15;15(51):59134–44. doi: 10.1021/acsami.3c12798 (PMC10755695; doi:10.1021/acsami.3c12798)
Supplement: Supplementary file 1 — am3c12798_si_001.pdf [file am3c12798_si_001.pdf]

Reaching the tumor: mobility of polymeric micelles inside an *in vitro* tumor-on-a-chip model with dual ECM

Alis R. Olea[1‡], Alicia Jurado[1‡], Gadi Slor[2], Shahar Tevet[2], Silvia Pujals[3], Victor R. De La Rosa[4], Richard Hoogenboom[4], Roey J. Amir[2,5,6,*], Lorenzo Albertazzi[1,7,*]

1 – Institute for Bioengineering of Catalonia (IBEC), The Barcelona Institute of Science and Technology, Baldiri Reixac 15-21, 08028 Barcelona, Spain

2 – Department of Organic Chemistry, School of Chemistry, Faculty of Exact Sciences, Tel-Aviv University Tel Aviv 6997801, Israel

3 – Department of Biological Chemistry, Institute for Advanced Chemistry of Catalonia (IQAC-CSIC), Jordi Girona 18-26, 08034 Barcelona, Spain

4 – Supramolecular Chemistry Group, Centre of Macromolecular Chemistry (CMaC), Department of Organic and Macromolecular Chemistry, Ghent University, Krijgslaan 281, S4, 9000 Ghent, Belgium

5 – The Center for Nanoscience and Nanotechnology, Tel-Aviv University, Tel Aviv 6997801, Israel

6 – The ADAMA Center for Novel Delivery Systems in Crop Protection, Tel-Aviv University, Tel Aviv 6997801, Israel

7 – Department of Biomedical Engineering, Institute of Complex Molecular Systems (ICMS), Eindhoven University of Technology (TUE), Eindhoven 5612 AZ, The Netherlands

[‡] These authors have contributed equally.

* Corresponding co-authors: l.albertazzi@tue.nl and amirroey@tauex.tau.ac.il

Supplementary figures

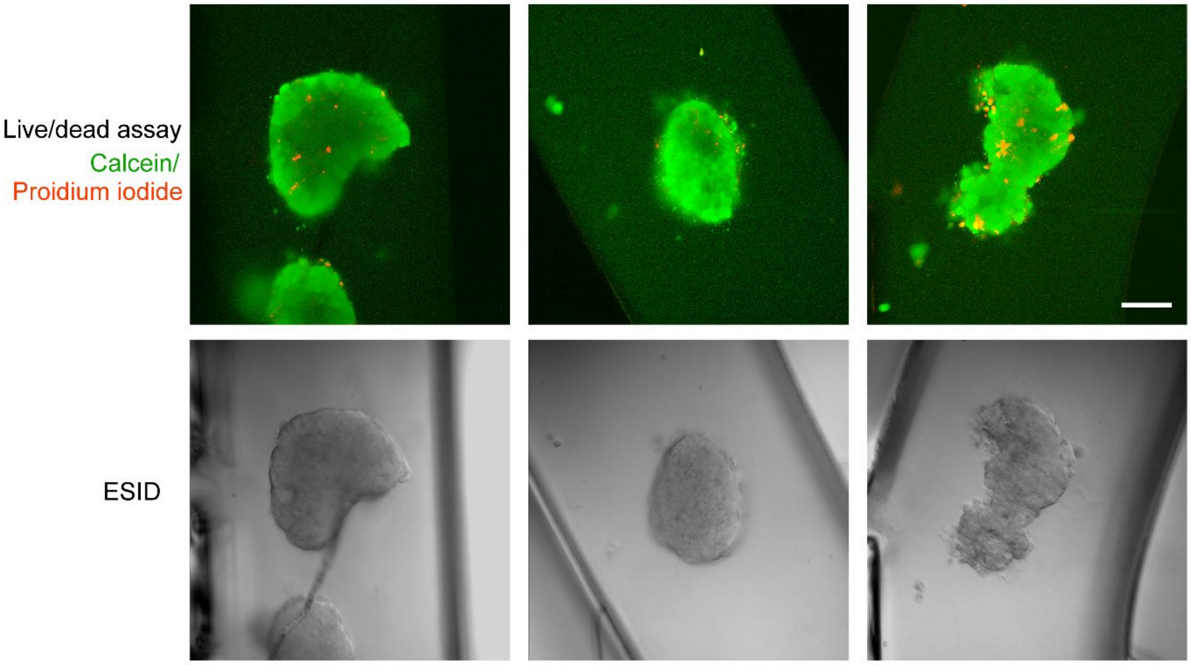

Figure S1: Viability of MCF7 spheroids after 24h inside the dual-ECM chip, assessed using a live/dead assay with calcein (green) and propidium iodide (red) respectively (n=4). Scale bar represents 100 µm.

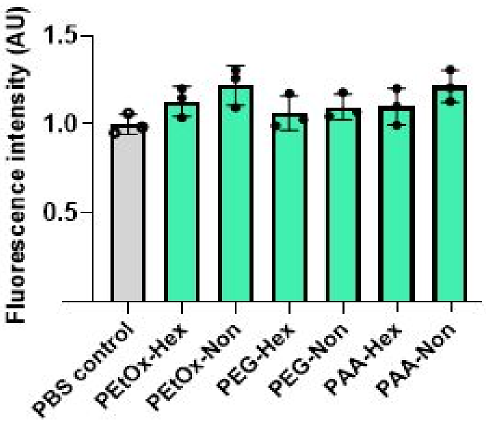

Figure S2: Cytotoxicity assay of micelles on MCF7 cells, assessed with Presto Blue (n=3).

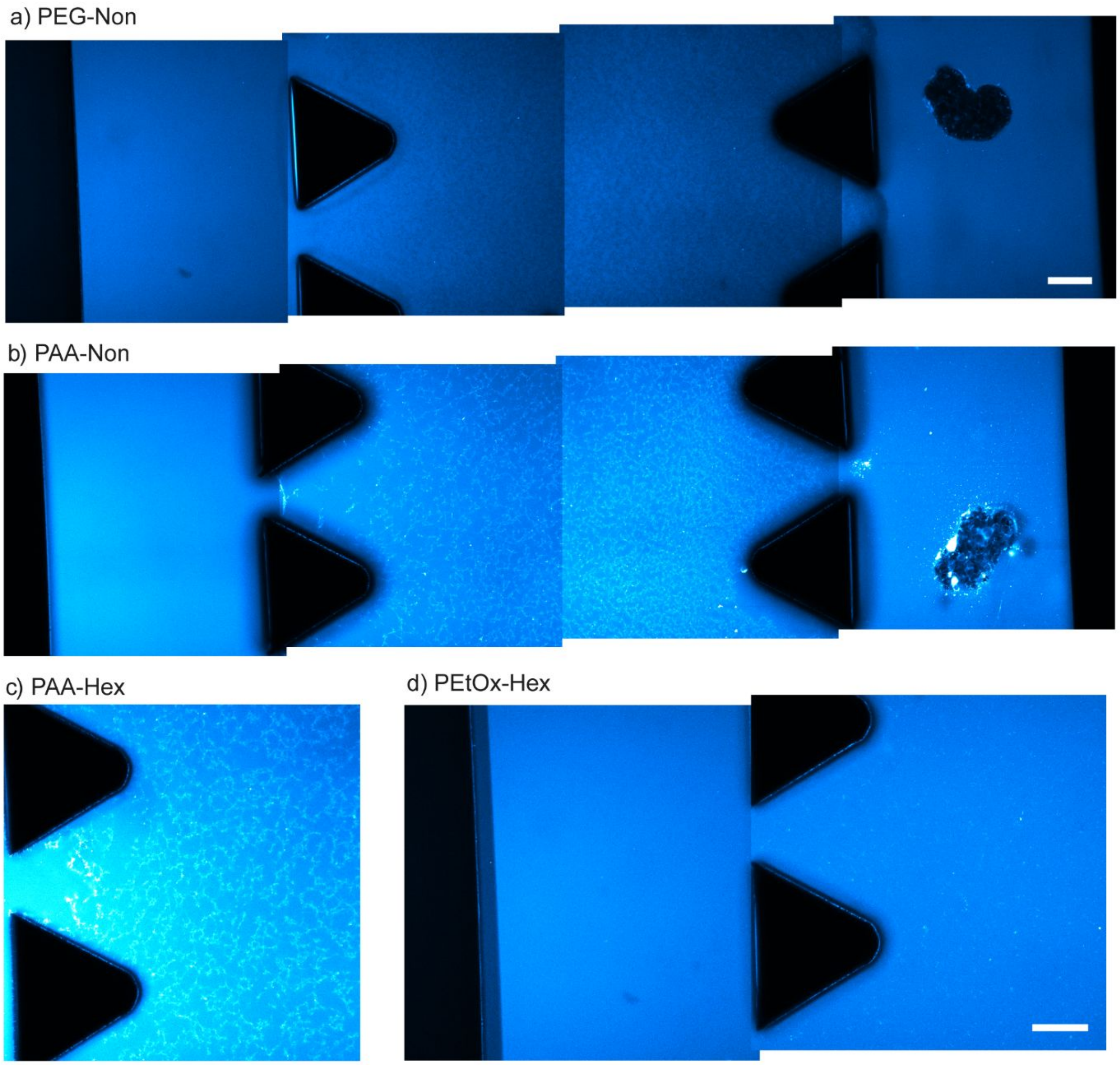

Figure S3: Overview images (total fluorescence) of coumarin-labeled micelles inside multigel chip model. The chip contains three channels separated by triangular pillars; the left channel contains only solution of micelles in full DMEM media, the middle channel contains a basal lamina gel model and the right channel contains MCF7 spheroids embedded in a mix of collagen type I and hyaluronic acid. The gel structure inside the middle channel was visible after micelle adition in some cases, either as a “dark” structure for more hydrophobic micelles (a – PEG-Non), or as a bright structure for PAA micelles (b – PAA-Non, c – PAA-Hex). In-between, the less hydrophobic micelles did not reveal the structure, but only a few bright spots when compared to the left channel (d – PEtOx-Hex). Scale bars represent 100 µm.

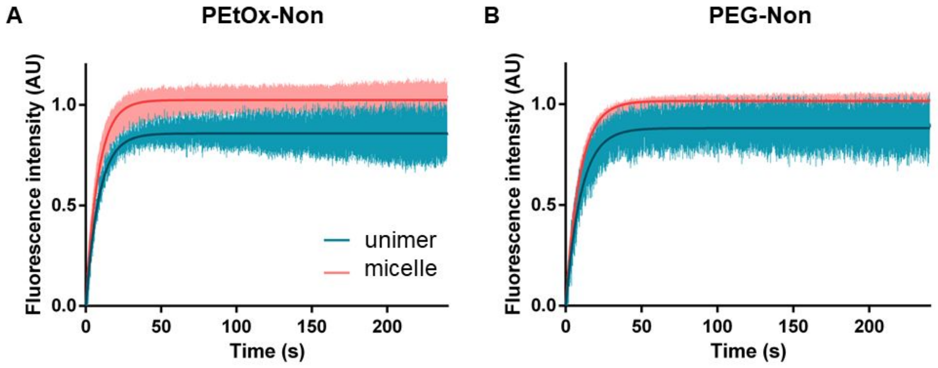

Figure S4: Zoom-out of FRAP recovery curves for PEtOx-Non (A) and PEG-Non (B) in collagen-HA next to spheroid, showing the mean fluorescence in the bleach area during the entire post-bleach acquisition time. Graph plotted as mean +/-SD. Unimer signal is shown in blue, while micelle signal is shown in red.

Supplementary methods

Cytotoxicity in 2D culture of MCF7 cells

MCF7 cells were seeded in a flat-bottom transparent 96-well plate (Nunc, Thermo Scientific) as 5000 cells/well and allowed to grow for 24h. The supernatant was replaced by 160µM micelle solution in full DMEM (10% FBS). PBS (pH 7.4) or Triton X-100 0.01% v/v in full DMEM were used as negative or positive controls respectively. After 24h, the cells were incubated for 1h with Presto Blue solution (ThermoFisher) as 10% v/v, at 37° C, 5% CO_2 . Florescence was measured using a multimode microplate reader (Infinite M200 Pro, Tecan), with 550 nm excitation, acquiring the signal at well bottom at 600 nm emission. Samples were prepared in triplicate, in randomized order.
